# The headcam mother-infant interaction assessment tool: testing the feasibility and acceptability in Soweto, South Africa, using participatory engagement

**DOI:** 10.1186/s40814-021-00875-3

**Published:** 2021-07-05

**Authors:** Stephanie V. Wrottesley, Rebecca Pearson, Alessandra Prioreschi

**Affiliations:** 1 SAMRC/Wits Developmental Pathways for Health Research Unit, Department of Paediatrics and Child Health, Faculty of Health Sciences, University of the Witwatersrand, Johannesburg, South Africa; 2 Centre for Academic Mental Health, School of Social and Community Medicine, University of Bristol, Bristol, UK

**Keywords:** Mother-infant interactions, Headcam, Acceptability, Feasibility, South Africa

## Abstract

**Background:**

Many children in low- and middle-income countries lack the stimulation needed to support healthy growth and development. Sensitive interactions between caregivers and infants may promote healthy movement behaviours in infants, which could improve childhood growth and development. However, reliable measures for such interactions require testing in the South African context. The aim of this study was to test the acceptability and feasibility of the headcam caregiver-infant interaction assessment tool in mothers from Soweto, South Africa.

**Methods:**

Nineteen mother and infant (6–24 months) pairs were asked to wear headcams (first-person observation) while participating in group and individual activities. Detailed instructions on headcam use were provided before and during these activities. Mothers were then asked to use the headcams, as well as photoframe cameras (which provided context of the interactions), in at least three, 5-min mother-infant engagement sessions at home over a 1-week period. Thereafter, focus group discussions (FGDs) were conducted to explore mothers’ experiences of using the tool in the home setting. The feasibility of the headcam mother-infant interaction tool was assessed according to a priori criteria which scored (i) technical reliability of the devices and (ii) usability of the recorded footage. Acceptability was assessed according to emerging themes which were coded from the FGDs using a constant comparison method by two researchers.

**Results:**

The headcam mother-infant assessment tool was found to be feasible in Soweto, and sufficient data was available to code. Three main themes emerged from the FGD analysis: use of the headcam, using the headcams in the home environment and using the photoframe vs. the headcam. Mothers remarked on the ease of using the tool across daily activities, the normality of their infant’s behaviour during recording and the acceptability by other members of the household. Large amounts of wasted unusable recordings were produced, and challenges related to switching the cameras on and off and to headcam placement were discussed.

**Conclusions:**

Our study shows that headcams are both an acceptable and feasible method for assessing mother-infant interactions in Soweto. However, improvements to the usability of the tool and the quality of the data collected should be made prior to future work.

**Supplementary Information:**

The online version contains supplementary material available at 10.1186/s40814-021-00875-3.

## Key messages regarding feasibility

- What uncertainties regarding feasibility existed prior to the study?
  - o While first-person observation has been used in some higher income settings, it is unclear whether headcam devices will be feasible for the observation of mother-infant interactions in South Africa.
- What are the key findings on feasibility from this study?
  - o The headcam devices were well accepted by mothers, as well as other family members, and proved feasible for use in this setting.
- What are the implications of the findings on the design of the main study?
  - o Adaptations to the devices can now be made to ensure usability is improved, and implementation of first-person observation can commence in South Africa.

## Introduction

Over half of South Africa’s children lived below the “upper-bound” poverty line in 2017—meaning that the minimum income requirement to meet their basic needs was not being obtained [1]. In addition, half of the country’s children live in densely populated communities, with poor living conditions (13% living in informal structures) and persistent unemployment [1, 2], resulting in an increased burden of care [3]. Thus, many children in South Africa are growing up in sprawling urban poor communities, where caregiving roles are traditionally less structured than in Western settings [1].

Nurturing care, which is a stable environment created by caregivers that ensures infant’s good health and nutrition, protects them from threats and gives young infants opportunities for early learning, through interactions that are emotionally supportive and responsive [4], is essential for optimal child development and growth. Specifically, effective caregiving interactions require sensitivity from the caregiver to detect the child’s signals and adequate responsiveness to meet his or her needs [5]. In settings of poverty, such caregiving relationships are all the more vital, as they promote the physical, intellectual and social development required by children to respond to challenges as they age [5]. However, in communities such as Soweto, nearly 50% of children under five grow up without their father in the home, and caregiving environments are not nuclear [1].

Within the framework of nurturing care, recent local and international movement guidelines prescribe that, even in the first 2 years of life, infants and toddlers should be provided with as much stimulation and opportunities to be active as possible in order to optimise development and growth [6, 7]. However, lack of stimulation in the home environment is common in South Africa, with data indicating that approximately a third of children are never read to or encouraged to imitate actions and sounds by their caregivers [2]. Sedentary behaviours and high screen time are also increasingly common, and accumulation of sufficient tummy time in young infants is rare [6]. Thus, many South African infants grow up in environments that do not provide opportunities for interactions or play to stimulate movement and learning, as well as healthy growth [1, 2]. This has important implications on the rising childhood obesity burden in South Africa, where a quarter of 2- to 5-year-olds are overweight or obese and, thus, at increased risk of becoming obese adolescents [8, 9]. Together, increasing obesity prevalence rates, poor adherence to movement guidelines [10] and a lack of stimulation highlight the need to explore measures of nurturing care and interactive stimulation in this context to inform interventions that promote early childhood development, thus, breaking the intergenerational cycle of poverty and ill health.

The first-person headcam measure has been validated and used to measure caregiver-infant interactions in higher income settings [11]. This technique has proven less invasive than other observation techniques [11] and has been shown to capture less biased interactions. However, it is possible that cultural, social and traditional factors in South Africa could impact the acceptability of first-person headcam use. Further improvements in the current methodology are also needed, with only 70% uptake of such measures in the UK (unpublished). It is thus likely that the protocol for using headcams will need to be modified for the South African context. It is therefore essential to engage participants in this process and to obtain their feedback in order to (i) develop a protocol that is feasible and acceptable and (ii) ensure the success of future observational or intervention studies using this methodology. The aim of this study is to test the acceptability and feasibility of the headcam caregiver-infant interaction assessment tool in mother-infant dyads in Soweto, South Africa.

## Methods

Twenty-seven women were contacted telephonically to participate in this study. Women were contacted from existing studies at the SAMRC/Wits Developmental Pathways for Health Research Unit (DPHRU) if they were >18 years old, if their child was aged between 6 and 12 months and if they lived in the Soweto area. Ultimately, 19 women arrived at DPHRU for their first visit (in three different groups of *n* = 5, *n* = 5 and *n* = 9). Data collection happened between May and July 2019. Women signed informed consent to participate in this study and to have their audio recorded, as well as to have their footage recorded from the headcams and shared with colleagues for analysis purposes. Ethical approval for this study was granted by the University of the Witwatersrand Human Research Ethics Committee (M190236). Women were able to request that any portions of their home recordings be deleted without being viewed by researchers and were given forms to record any dates and times of applicable videos. They were also advised to explain the use of the headcams to other family members and were reassured that any footage showing family members could also be deleted upon request, and if not deleted, footage of other family members would not be coded or shared. Women and infants were provided with refreshments during each visit and were reimbursed for their travel costs.

### Participatory engagement sessions

**Fig. 1:**
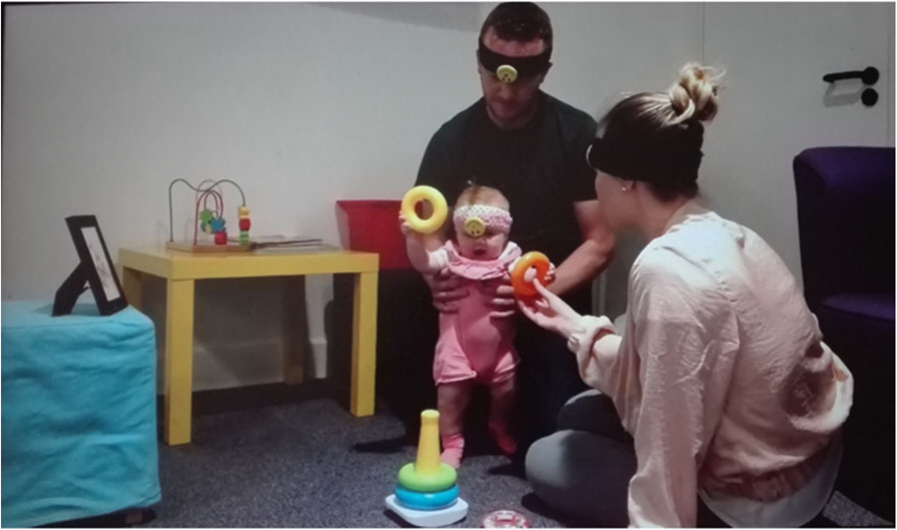
An example of caregivers in the UK using the photoframe camera (left) and the headcams (attached to infant and parents)

In order to engage with participants around practical issues regarding the use of headcams in this population, women initially attended one half-day session with their infants at DPHRU. Demographic information, such as age, education, socioeconomic status, employment and child’s age and sex, were recorded at the beginning of the session. Mothers and their infants were then asked to wear headcams attached to a soft fabric headband while participating in both group and individual activities. Group activities included play circles, mother-infant dancing, free play and feeding. Before and during these activities, caregivers were instructed on how to use the headcam devices and were asked to initialise them. Women were also shown how to use the photoframe cameras (which capture the interaction from a third-person perspective, Fig. 1). After each play activity, women viewed their footage in order to see what was being captured from both perspectives and in order to determine if adjustments to the positioning of the cameras were needed.

Thereafter, women were asked to take the headcams and, in some cases, the photoframe devices home and attempt to use them in at least three, 5-min interaction sessions of their choice with their child over a 1-week period (examples such as feeding, playing and changing were provided). Mothers were given instruction forms to take home to remind them how to use the headcam and photoframe devices (Supplementary File 1) and were asked to record the times at which they performed these interactions using a diary.

### Focus group discussions

One week after attending the participatory engagement sessions, mothers were asked to return the devices and to participate in a focus group discussion (FGD). During the three FGDs (one for each group of women), a semi-structured interview guide with a relatively flexible framework for discussion was used (Supplementary File 2). The interview guide focussed on the acceptability of the headcams in the home setting, as well as the feasibility of their use. Mothers were asked to report on any realised or foreseeable barriers for using the headcams and any concerns they had. They were also asked to reflect on practical issues that arose, including (but not limited to) when they could use the device for recording at home (i.e. during feeding, playing), the positioning of the device, the duration and frequency of recording, how easy the device was to use and perceptions of other family members within the home. All FGDs were recorded and transcribed verbatim.

### Data analysis

#### Feasibility

In order to assess the overall feasibility of the headcam mother-infant assessment tool, video footage from the headcams, and photoframes where applicable, were downloaded and viewed for each mother-infant dyad. Technical reliability per device for each mother-infant dyad was evaluated by recording whether all videos for a mother-infant dyad were successfully downloaded and viewed. Thereafter, usability of the headcam and photoframe footage per mother-infant dyad was determined by classification according to the following criteria:

(i) At least two corresponding videos of ≥ 2 min were recorded from the mother’s and the infant’s headcam devices
(ii) At least one interaction of usable quality (the mother or the infant, or both in the case of the photoframe, were clearly visible on the videos) was collected for the mother-infant dyad
(iii) No video recordings for a mother-infant dyad were requested to be deleted.

For both technical reliability and usability, the tool was deemed feasible if at least 80% of mother-infant dyads (i.e. 16/19 for the headcams and 13/15 for the photoframes) met the technical reliability and usability criteria.

#### Acceptability

Acceptability of the headcam mother-infant assessment tool was assessed using qualitative analysis of the FGDs. Specifically, FGD transcriptions were read and coded by two researchers who used a combination of deductive (pre-identified themes based on the research question) and inductive (emerging themes from the transcripts and field notes) approaches to identify and analyse themes. Thereafter, transcripts and coding frameworks were cross-checked for interpretation and theme identification. Researchers provided each other with individual reviews of the coding frameworks, where after meetings were held to compare, contrast and discuss emerging themes, while incorporating the principles of the immersion-crystallisation method (whereby researchers engage with the data in detail, and then take time to reflect on the patterns and themes). A separate researcher then tested the reliability and internal validity of the data coded from the FGDs, and, where differences were established, these were discussed and resolved as a group.

Common and unique themes across the three transcripts were collated to develop an overall data codebook for the three FGDs. Following coding, a group meeting was held to complete data analysis and interpretation of themes, and the codebook was interrogated and refined until the point that no new themes emerged. In order to illustrate the themes and subthemes that emerged from the FGDs, exemplar quotations were excerpted. Finally, data were presented according to the main themes that emerged.

## Results

The mean ± SD age of mothers was 34 ± 4 years and that of their infants was 11 ± 2 months. Two-thirds of the infants were male. The majority of mothers were single (70%) and unemployed (90%).

### Feasibility

As summarised in Table 1, the headcam mother-infant assessment tool was found to be feasible in Soweto. Specifically, the technical reliability of the tool was good, with all videos from the mothers’ and infants’ headcams being downloaded and viewed successfully. For the photoframe devices, only one video from one mother-infant dyad could not be opened for viewing after it was downloaded from the device. The usability of the video footage was good overall, with >80% of mother-infant dyads recording at least two videos per device of usable quality. No mothers requested that any video interactions from the headcams or the photoframe devices be deleted prior to viewing.

**Table 1:** Feasibility of using the headcam mother-infant interaction assessment tool in Soweto, South Africa

Criteria	Mother-infant dyads meeting criteria	
	***n***	***%***
**Technical reliability**		
All videos from the infant’s device successfully downloaded and viewed	19/19	100
All videos from the mother’s device successfully downloaded and viewed	19/19	100
All videos from the photoframe device successfully downloaded and viewed	14/15	93
**Usability of footage collected (headcam,** ***n*** **= 19)**		
At least two videos of ≥ 2 min each recorded for mother-infant dyad	16/19	84
At least one interaction of usable quality collected	17/19	89
No video interactions for mother-infant dyad requested to be deleted	19/19	100
**Usability of footage collected (photoframe,** ***n*** **= 15)**		
At least two videos of ≥ 2 min each recorded	13/15	87
At least one interaction of usable quality collected	14/15	93
No video interactions requested to be deleted	15/15	100

Feasibility assessed according to the number of participants meeting the criteria. A total of at least 16/19 for the headcam and 13/15 for the photoframe (i.e. >80%) was considered feasible

However, while the criteria for reliability and usability were met and sufficient data was available to code for >80% of participants, unusable data was also recorded in addition to the useable data obtained, with mother-infant dyads recording on average four (range 1–12) unusable headcam and two (range 1–5) unusable photoframe videos. Criteria that resulted in videos being unusable in order of prevalence were (1) footage was too short (i.e. the camera was switched on and immediately off, usually by mistake), (2) devices were poorly positioned, (3) footage was too dark, and (4) there was excessive movement during interactions and/or mom and/or infant were not visible. This resulted in a lot of wasted participant and researcher effort recording and assessing unusable videos, which indicates the need to improve the quality of footage for ease of use in future work. These devices should specifically allow for more accurate positioning that can be verified by participants in real time, as well as a wider range of view for recording.

### Acceptability

Three main themes emerged from the FGD analysis: use of the headcam, using the headcams in the home environment and using the photoframe vs. the headcam. Some of these themes were further divided into subthemes.

### Use of the headcam

#### Ease of use

Mothers reported that, while it initially took time to get used to the headcams, they generally enjoyed using the cameras and had no major issues with doing so. The main issue reported with using the headcams was that they do not have specific “on/off” indication lights, so mothers found it difficult to know if they had switched the camera on correctly.

"… I didn’t know when it is on and when it is off". FGD 1

Mothers reported that the instruction sheets given to them were useful and helped them to follow the process at home as they could re-read the instructions if they were unsure.

#### How babies acted

Some mothers reported that the placement of the headband on the baby’s head was “irritating” and “uncomfortable”. Mothers specifically mentioned their infants wanting to remove the headbands and play with them, or the headbands being too big for very small babies and therefore slipping down. Mothers mentioned that the forehead may not be the best location for the camera.

"The head band I think that is what is uncomfortable for the kids. So, maybe if you can put it on the tummy somewhere where they will not be playing with it. Any time you put on the head they want it off". FGD 1

The majority of mothers stated that the infants just wanted to play with the headcam and thought it was a toy. It seemed that it was easier to use the headcams with some babies than with others, as some mothers stated that their babies were happy with the headband placement. Besides changing the location of placement of the headcam, mothers did not provide any other suggestions for improving the ease of use of the camera.

The majority of mothers reported that they did not feel their infants acted differently when wearing the headcams (besides wanting to play with the camera initially), either stating that their infant was “acting fine” in one case or was “just herself” in another. However, one mother reported that her son did not like the camera and was “uneasy”, while another mother reported that she doubted we would get any footage as her infant “didn’t want anything… he was just taking it off”. One mother also stated that using the camera changed where her child would normally play, as she did not want to let her child play outdoors with the camera on for safety reasons.

"For me it was a bit difficult because she likes to hold things, doesn’t want anything on her head. So, for me it was very difficult because she is difficult, she likes to play outdoors most of the time. So, when we put on the camera she didn’t go outside. Because it needs to be staying with her". FGD 1

#### Best time or activities to record

Some mothers reported that it was difficult to record while their infants were playing as their infants would move around a lot. While some mothers found play time easy to record, the majority reported that recording during feeding time was the easiest. However, other mothers found it difficult to record during feeding and preferred to do so during the early morning or during the afternoon.

"Feeding time was when I sat face to face, play time she would run around everywhere". FGD 1

"With feeding time we fight, feeding time was terrible". FGD 1

Therefore, there was no consensus on the best activity to record or the best time to record; however, the mothers all stated that it was easy for them to find a time that worked for them and that they preferred to use the camera when other people were not around as this would cause distractions.

"I would play with my baby and if they saw someone else in the kitchen he was going to run to them". FGD 2

### Using the headcams in the home environment

Mothers stated that they felt comfortable using the headcams in their home and did not report any barriers. However, some mothers felt uncomfortable under certain circumstances or when doing certain activities. They also discussed not always being sure which activities to do when recording, or being unsure of what they were “allowed” to do on camera.

"Sometimes I want to record her when she is sleeping. I didn’t know if you would want that ... I’m not sure if it would be appropriate for you guys". FGD 2

"I was a bit scared because I normally feel the bond when I was breast feeding, I didn’t know. The eye contact while feeding. I really feel the bond". FGD 2

"Because for me … if she is bathing then we interact better. I recorded once when she was bathing but eish maybe she put it [the headcam] into the bath". FGD 2

One mother suggested giving more examples of activities in the instruction form.

"I think with activities because I ran out of activities because we didn’t do a lot I didn’t know what to do. So far I recorded her eating, playing and that was it. Maybe other activities". FGD 2

#### Family members’ perspectives on the use of the headcam

The majority of mothers reported that other family members or people in the home did not have a problem with the use of the headcam. In fact, mothers reported on how other family members also wanted to be in the recording.

"Because … on Thursday when I came with it sjoe* everyone wanted to be on video". FGD 1

*Sjoe is commonly used emotive South African slang roughly translating to “wow”

"…I have a 10 year old son he just wanted to pass this like the one that is younger.

Interviewer: He wanted to be on camera?

Ja, he wanted to do that so he was passing [in front of] the camera... " FGD 2

A small number of mothers reported that other people in the home questioned what the cameras were for, but found that they were able to explain this easily. Thereafter, family members were happy with the use of the headcams in the home and were aware of how the data would be used. As shown by the feasibility assessment, no mothers requested that we delete any specific videos from the headcams or photoframe devices.

"My sister asked me what are you going to do with it. Then [I said] because you want to see how we bond with the baby and then they were okay". FGD 1

One mother reported that her sister was not happy being on camera because she would not “appear nicely” and was presumably worried about her appearance. Another mother also reported that her husband mentioned that everyone would now “…see that you have depression”. A few people who saw the headcams apparently asked to buy them. One mother wanted to keep the headcam to send with her child to creche to observe what was going on.

"… this one is good I can take it to my child’s crèche…please just leave it like this I want to see something ... "FGD 2

### Photoframe vs. headcam

The mothers unanimously agreed that the photoframe was better than the headcam. Photoframes were not given to all mothers due to limited availability, but mothers consistently mentioned that it would be better if they could either have the headcam switched on and recording all day, or simply use the photoframe and leave it recording. This was seen as easier than having to remember to switch the headcam on and off.

"I felt like why not switch on and leave it there.

Interviewer: So, to film everything?

Just leave it there to record everything ... "FGD 1

"Also I think it would have been better if it was recording the whole morning, or afternoon or the whole day. You know when you get kids to play with you they sometimes want to do their own stuff". FGD 2

"And you want them to concentrate on what you are doing it is difficult. Then it is better if you are recording the whole day because then you have those moments whereby you are alone the two of you and you wish you had captured that". FGD 2

"For me I think it is better to be on for the whole day because sometimes you [forget], he would be crying and you want to switch it on or off, he is crying, you can’t". FGD 3

However, the point was raised that with older infants who were more mobile, the photoframe would not work as they would not stay in one place.

"When the baby is young like 6 months then it is okay. When the baby is crawling, then the baby goes outside you never take the frame and follow him, it is so difficult". FGD 3

## Discussion

The aim of this study was to test the acceptability and feasibility of the headcam mother-infant interaction assessment tool in Soweto, South Africa. To our knowledge, this is the first study to explore using wearable cameras to assess mother-infant interactions in Africa. Overall, our data showed that mothers found it both acceptable and feasible to use the devices in their home environment, specifically remarking on their ease of use across daily activities, the normality of their infants’ behaviour during recording and the acceptability by other members of the household. However, our findings also highlighted modifications that should be made to improve headcam usability, as well as the quality of data collected, in future studies. In particular, attention should be given to how the cameras are turned on and off, as well as how this is indicated on the device, and to headcam placement.

Since recognition of the important roles that nurturing care environments and responsive caregiving interactions play in facilitating healthy infant growth and development, the use of appropriate and reliable methods for measuring mother-infant interactions has received increased attention. While studies have traditionally made use of third-person observations or recordings by researchers, such methods are limited in that they can be perceived as intrusive, are based on a subjective viewpoint and are restricted by researcher and participant time [11]. For these reasons, wearable first-person headcams, able to simultaneously record the perspectives of caregivers and their infants, are being increasingly utilised in higher income settings [11–13]. Such devices have been shown to reliably capture interactions when compared with third-person recordings, while allowing for greater recording durations, more subtle detections of facial expressions and vocalisations, and a reduced burden on researcher time [11].

It has been suggested that—despite the absence of a third person—the presence of the headcam itself may influence caregiver and/or infant behaviour, thus, providing only a partially representative view of mother-infant interactions [12]. While important to consider, parents in previous studies have reported forgetting about the presence of the camera—suggesting acclimatisation to the headcam and an increased likelihood of usual behaviour [12]. The use of headcams has also been shown to provide rich and variable data from the normal environment in which caregivers and infants interact, alongside more accurate representation of the infant’s perspective compared to similar studies which use only an adult’s perspective [12]. During our study, mothers reported that, for the most part, their infants acted normally when using the headcams. Where changes in behaviour were noted, these were predominantly related to discomfort in the positioning of the headcams—usually when the headband holding the camera was too big for the child’s head or the child was not used to wearing head accessories—or their infants were distracted by playing with the devices. Similarly, while some mothers expressed uncertainty in what activities they should, or were allowed, to do on camera, the majority described being comfortable including the cameras in daily interactions with their infants, and most used these while either feeding, playing or dancing with their infants. This suggests that the presence of the cameras themselves had little influence on mother-infant interactions and that the recordings would largely represent normal behaviour, especially as infants started to get used to them. In addition, through addressing more practical concerns about headcam use—particularly modifying headcam placement and/or comfort, and providing clearer instructions to moms about when and what to record—we could ensure that recorded interactions are even more representative of daily life.

Due to the absence of evidence on wearable camera use in low- and middle-income countries, understanding how environment and culture may influence the acceptability of these devices within community and home environments is critical to planning future research. Even in high-income settings, studies which explore the acceptability of videotaping for research purposes are limited—with assessment of videotaping predominantly focused on documenting primary healthcare consultations for training purposes [14]. However, a review of such studies suggests that, while many patients/participants understand the value of videotaping and are happy to take part, concerns around ensuring that privacy, dignity and safety are maintained throughout the research process may limit participation in some cases [14]. Within the home, such concerns may be exacerbated by the potential for other household members to be recorded—without appropriate understanding of the research or informed consent protocols being in place. Previous studies suggest that in low- and middle-income settings, where distrust and cultural taboos around videotaping may exist, recording interactions may be even more challenging [15]. However, our findings show that, in Soweto, the use of cameras is an acceptable method of assessing mother-child interactions in the household. Other family members in the home did not express concern at possibly appearing in the footage; however, considerations of data protection, confidentiality, consent and sharing are important when using recording devices in the home, specifically when children are involved [16]. We believe that our inclusion of dialog with mothers in these development and piloting stages is essential. Furthermore, regular open dialog with ethical committees is crucial [16].

While mothers were willing to use both the headcams and the photoframe cameras at home, there was a strong preference for using the photoframe which removed issues around wearability for the infant, as well as the need to turn the cameras on and off for each use. This is an important preference to acknowledge, as it reinforces some of the ways in which the headcams can be made more user-friendly and highlights the acceptability of including a third-person perspective camera in future research. While the photoframe does not allow researchers to directly perceive interactions from the caregivers or the infant’s perspective, we have found that it provides a valuable overall view of the interaction within the wider home context, and if positioned well still allows for analysis of many of the interaction domains of interest.

### Limitations

While our study allowed us to test the feasibility and acceptability of using the headcam tool in Soweto, our sample was limited to mother-infant dyads. Thus, the applicability of our findings to other caregivers, such as grandmothers and fathers, who play a central caregiving role in this context, was not assessed. In addition, although mothers reported that other family members were happy with the use of cameras in their homes, we did not assess this from the family members’ perspectives. Thus, ensuring that the tool is feasible and acceptable across different caregivers and including other family members in discussions around camera use within the home would be beneficial and would inform the potential for wider use of the tool. The majority of the women included in our study were unemployed and therefore may have had comparatively more time available to record interactions than a sample of employed women would have. While this is representative of the Soweto population—where unemployment rates are high—it is not clear from our data whether this method would be feasible and acceptable to caregivers who are employed, and this should be explored.

### Future directions

While this study shows that the headcam mother-infant interaction tool is a feasible and acceptable method of assessment in Soweto, South Africa, it highlights necessary modifications to improve usability in future studies. Specifically, age-appropriate or adjustable headbands should be used in order to ensure that headcams fit comfortably—particularly at younger ages. While the positioning of the camera on other areas of the infants’ body has been considered, development work has shown that the head provides footage most representative of first-person vision, being just slightly above the eyes. In addition, more detailed instructions on when and what to record should be given to mothers (and other caregivers where applicable), while still encouraging them to continue daily interactions with their infants as normal. Finally, headcams that more clearly indicate on and off modes, signal when the device is successfully recording and allow for a wider view in the camera lens should be used. This has been undertaken by the authors and collaborators, through the development of an improved headcam device, which addresses issues raised by researchers and participants from low- and high-income settings globally. Specifically, the new device has a more secure and comfortable attachment, a clear on/off button, a wider range of view and clear indicators for function.

## Conclusion

In conclusion, our study shows that headcams are both an acceptable and feasible method for assessing mother-infant interactions in Soweto. This provides an important foundation for the use of camera-based methods to objectively assess the role that caregiving interactions have in promoting early childhood growth and development in future studies. However, it also highlights where the usability of the tool and the quality of the data collected can be improved prior to future work.

## Supplementary Information

**Additional file 1.** Instructions for using the Headcam

**Additional file 2.** Baby headcam acceptability and feasibility in Soweto Focus group discussion guide

## Data Availability

The datasets used and/or analysed during the current study are available from the corresponding author on reasonable request.
