# Supplementary material for: The headcam mother-infant interaction assessment tool: testing the feasibility and acceptability in Soweto, South Africa, using participatory engagement
Source: Pilot Feasibility Stud. 2021 Jul 5;7:140. doi: 10.1186/s40814-021-00875-3 (PMC8256612; doi:10.1186/s40814-021-00875-3)
Supplement: Supplementary file 1 — Additional file 1. Instructions for using the Headcam [file 40814_2021_875_MOESM1_ESM.docx]

Instructions for using the Headcam

**Step 1: Turn on the headcam**

Press the power button for 3 seconds until an orange light shows.


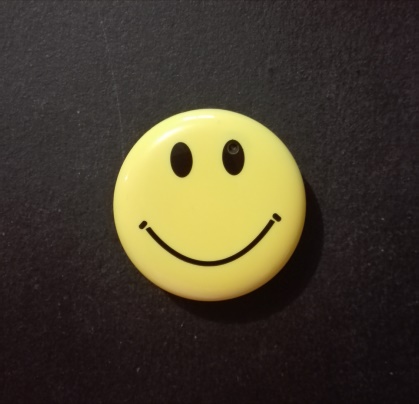

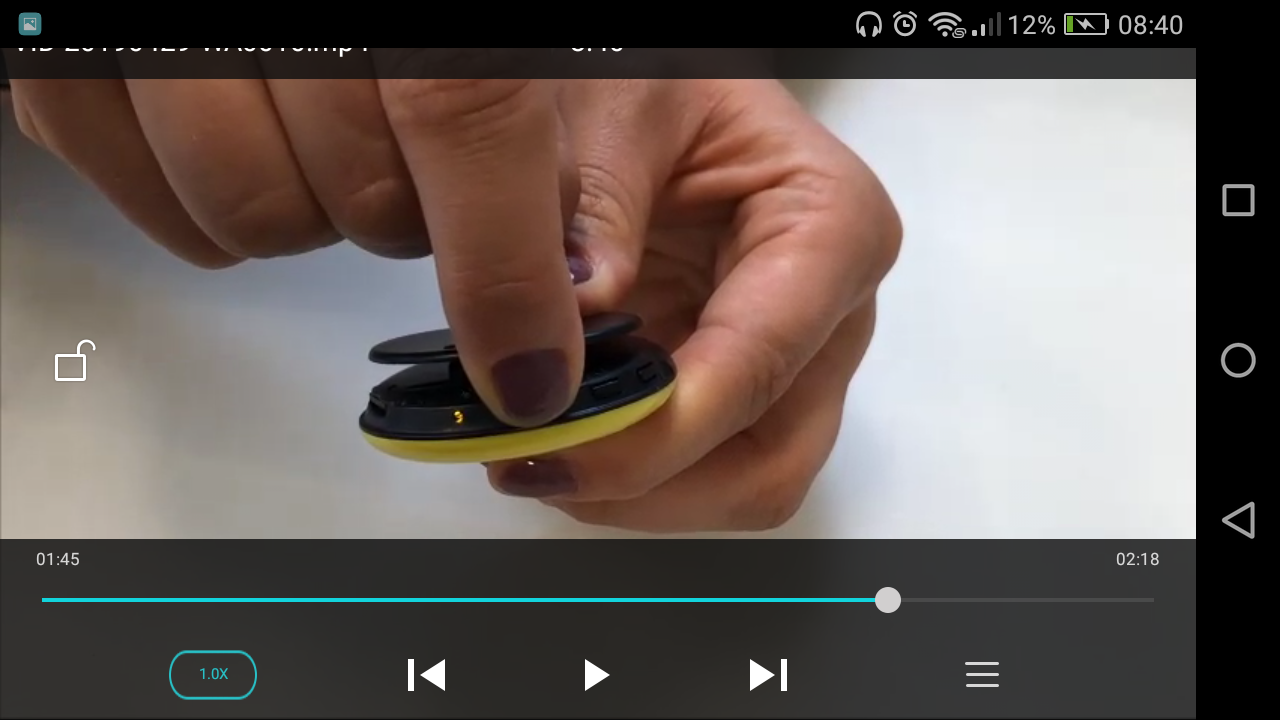


**Step 2: Start recording**

Press the middle button for 3 seconds until the orange light disappears. **The camera is now recording!**

**Step 3: Wear the headcam**

Click the headcam onto the headband and position the headband on your head so the camera is in the center of your forehead facing downwards. The headband must be as low down as is comfortable for you and sit just above your eyes so that your eyebrows are covered.


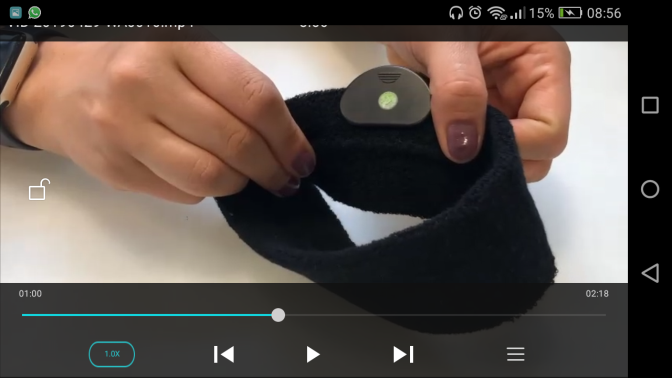

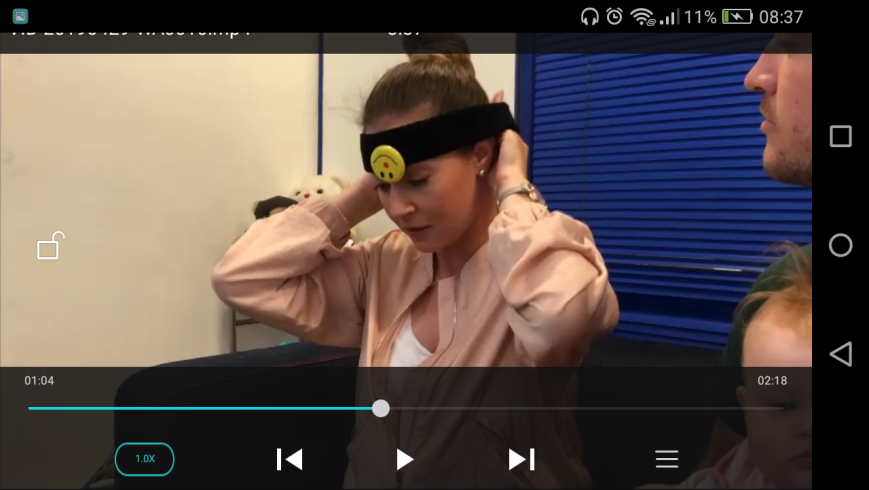


**Step 4: Turn on your child’s headcam**

Turn the headcam on in the same way as described above. Click the headcam onto the headband and position the headband in the same way – with the camera facing downwards in the center of your child’s forehead and the headband covering his/her eyebrows.


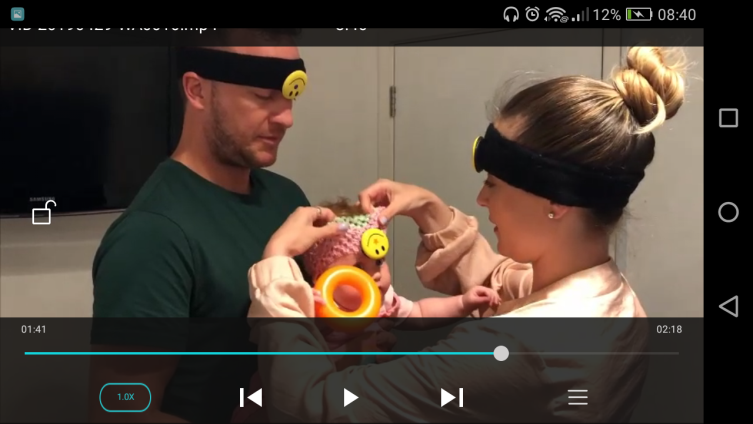

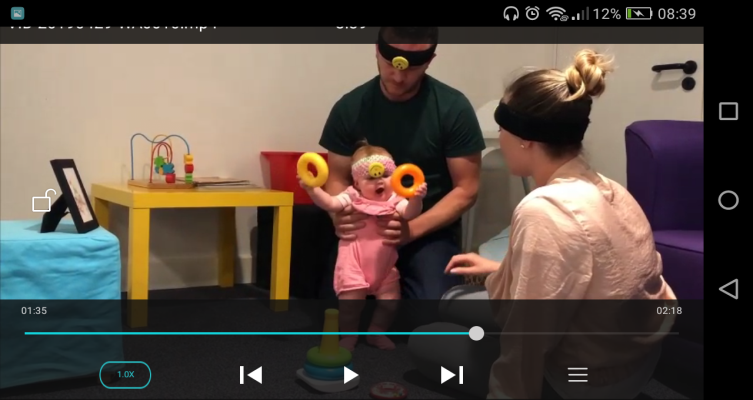


**Step 5: When you have finished recording**

Remove the headband and turn off the headcam by pressing the power button for 3 seconds until an orange light shows.

The video footage will be saved automatically to the headcam.
