# Supplementary material for: The headcam mother-infant interaction assessment tool: testing the feasibility and acceptability in Soweto, South Africa, using participatory engagement
Source: Pilot Feasibility Stud. 2021 Jul 5;7:140. doi: 10.1186/s40814-021-00875-3 (PMC8256612; doi:10.1186/s40814-021-00875-3)
Supplement: Supplementary file 2 — Additional file 2. Baby headcam acceptability and feasibility in Soweto Focus group discussion guide [file 40814_2021_875_MOESM2_ESM.pdf]

**Baby headcam acceptability and feasibility in Soweto**

**Focus group discussion guide**

## Purpose of the focus group:

To explore how mothers in Soweto, South Africa, experience the use of headcams to capture caregiver-infant interactions within their homes. This discussion will focus on the acceptability and feasibility of using the headcams with their infants (12 months of age) over the previous week in order to optimise use of this tool in future research.

**Date: ______________________ Starting Time: _________ Ending Time: ___________**

**Please fill out and attach the participant register.**

**Name of Facilitator: _______________________________________________________**

**Name of Note Taker: _____________________________________________________**

**Name of Observer: ________________________________________________________**

**DIRECTIONS:** The qualitative focus group begins with open-ended questions that point to starting a dialogue. The interviewer should encourage the study participants to do most of the talking but should use the questions listed here as a guide. After you ask each question, wait for the study participants to respond and go on to the next question when you are satisfied with the answer. If it seems as though the study participants did not understand a question, then repeat it or ask it in another way. If the study participants go on talking without much prompting, then let them guide the conversation. Bold indicates major questions and probes are in parentheses or listed below. Mentally check off these questions as they are asked so you do not repeat a question if it has been discussed previously.

**Remember to audio record each focus group. Use a backup recorder to prevent technological problems.**

**Note to the Facilitator:**

**Introduce yourself at the beginning of the session, explain who you work with, why you are here, and introduce everyone on the team who is with you observing, taking notes, or helping in anyway.**

**Introduction:** Hello, my name is ________________________________. I am working with researchers at the Developmental Pathways to Health Research Unit (DPHRU) to develop a study on caregiver-infant interactions and the influences on infant movement, play and feeding, as well as growth and development. We are interested in getting your views on using headcams with your

babies at home over the last week. We would like to ask you some questions about your experiences of using the headcams, how this made you (and other family members) feel, and any problems you may have experienced. This should not take more than one hour. Do not worry; there are NO right or wrong answers. Your experiences and ideas are very important to us and you should feel free to express whatever you are thinking. Your responses are confidential and will not be shared outside of this group. Please feel free to ask for clarification if you do not understand a question

**(Introduce the others on the team)** Do we have your permission to continue?

**Note to Note-Taker**: Try to capture the major ideas and always note the specific question that the facilitator and participants are referring to. If the facilitator asks a question that is not on the guide, note the question as it is asked and try to capture the answers.

***Acceptability and feasibility of using the headcams:***

1. **How was your experience of using the headcams with your babies over the past week?** (Free discussion about how the participants felt using the headcams)
    a. Probe: How did they feel using the headcams with their babies?
    b. Probe: Were the headcams comfortable to wear?
    c. Probe: Did they feel comfortable using the headcams in their own homes?
    d. Probe: Did they feel that using the headcams made them change the way in which they interacted with their baby at all?

2. **How do you think your baby felt wearing the headcam?**
    a. Probe: How comfortable do they think their baby was wearing the headcam?
    b. Probe: Did the headcam fit well and sit comfortably on the baby's head?
    c. Probe: From their experience of their baby, do they think the baby interacted naturally when wearing the headcam?

3. **How was use of the headcam viewed by other members of the household?**
    a. Probe: Did they feel comfortable using the headcam with their baby if there were other family members present?
    b. Probe: Did anyone in the household express concerns over use of the headcam in the home?
    c. Probe: Did anyone in the household express concerns about the possibility of being seen/ recorded by the headcam when it was in use?

4. **Did you fully understand how and when the headcams should be used?**
    a. Probe: Was there anything that could have been better explained about why they were using the headcams?
    b. Probe: Was there anything that could have been better explained about how to use the headcams?
    c. Probe: Was there anything that could have been better explained about when to use the headcams?

5. **When did you find it was best to use the headcams?**
    a. Probe: During which activities did they find it easier or more difficult to use the headcams?
    b. Probe: What time of the day did they find it was best to use the headcams?
    c. Probe: Did anything within the household prevent them or make it more difficult for them to use the headcams?

6. **Did you experience any difficulties when using the headcams?**
    a. Probe: Were there any barriers to effectively using the headcams in their home?
    b. Probe: Were the headcams easy to use and did they work properly?

***Views around parenting and caregiver-infant interactions:***

7. **How many hours per day do you spend caring for your baby?**
    a. Probe: When they are not caring for their baby, who usually looks after him/her?
    b. Probe: Who feeds the baby most of the time?
    c. Probe: Who plays with the baby most of the time?
    d. Probe: Who puts the baby to bed most of the time?

8. **How do you understand your role in the growth and development of your baby?**
    a. Probe: Do they think the way in which they interact with their baby is important for his/her development?
    b. Probe: Do they think the way in which they talk to your baby is important?
    c. Probe: Do they think playing with their baby is important?
    d. Probe: How do you they think how and what they feed their baby is important for their growth and development?

9. **What support do you have in caring for your baby?**
    a. Probe: Do they have any sources of information the assist them in adequately caring for their baby
    b. Probe: Do they have physical support from family or friends in caring for the baby?

***Final questions/comments:***

10. **Are there any other experiences or feelings that you would like to express about the headcams or their use?**

11. **Do you have any other questions you would like to ask us?**
